# Evaluation of Intestinal Absorption of Dietary Halocynthiaxanthin, a Carotenoid from the Sea Squirt *Halocynthia roretzi*

**DOI:** 10.3390/md18120588

**Published:** 2020-11-24

**Authors:** Chiaki Ikeda, Yuki Manabe, Nami Tomonaga, Tatsuya Wada, Takashi Maoka, Tatsuya Sugawara

**Affiliations:** 1 Division of Applied Biosciences, Graduate School of Agriculture, Kyoto University, Kyoto 6068502, Japan; ikeda.chiaki.58x@kyoto-u.jp (C.I.); manabe.yuki.8c@kyoto-u.ac.jp (Y.M.); tomonaga.nami.52v@kyoto-u.jp (N.T.); 2 Nihon Pharmaceutical Co., Ltd., Tokyo 1030012, Japan; tatuya.wada@nihon-yakuhin.co.jp; 3 Division of Food Function and Chemistry, Research Institute for Production Development, Kyoto 6060805, Japan; maoka@mbox.kyoto-inet.or.jp

**Keywords:** halocynthiaxanthin, intestinal absorption, carotenoid, functional food, metabolism

## Abstract

Halocynthiaxanthin is an acetylenic carotenoid mainly found in *Halocynthia roretzi*. To date, several bioactivities of halocynthiaxanthin have been reported, but its mechanism of digestion and absorption in mammals has not been studied yet. In this study, we evaluated the intestinal absorption of halocynthiaxanthin in mice. The halocynthiaxanthin-rich fraction was prepared from the tunicate *Halocynthia roretzi*. Mice were orally administered the fraction at a dose of 5 mg/kg body weight. The halocynthiaxanthin levels in the plasma, liver, and small intestine, were quantified using HPLC-PDA, 1, 3, 6, and 9 h after ingestion. The halocynthiaxanthin-rich fraction mainly consisted of the all-*trans* form and a small amount of *cis* forms. These three isomers were detected in the plasma of mice 3 h after ingestion. Time-course changes after the ingestion of this fraction were found, with *cis* isomers being more abundant than the all-*trans* isomer in the mouse plasma and liver. In the small intestine, however, the all-*trans* isomer was primarily detected. The possibility that *cis* isomers might be absorbed rapidly from the small intestine cannot be denied, but our results suggest that dietary all-*trans*-halocynthiaxanthin might be isomerized to the *cis* isomer after intestinal absorption.

## 1. Introduction

Carotenoids are a family of yellow to red lipid-soluble pigments with a long chain of conjugated double bonds. They are widely distributed in nature, and more than 800 members have been identified so far [1,2,3]. Microorganisms and plants biosynthesize carotenoids; however, most animals cannot synthesize carotenoids on their own. Animals ingest carotenoids from food; thus, accumulated dietary carotenoids and their metabolites can be found in animals [3]. To date, a variety of bioactivities of carotenoids have been reported and have attracted the researchers’ attention. In particular, some marine carotenoids exhibit strong bioactivities due to the unique chemical structure of carotenoids which are not found in terrestrial organisms. For example, fucoxanthin, which was identified from seaweed, that is traditionally eaten in Japan, such as kombu and wakame, shows strong anti-obesity and anti-inflammatory effects [4,5]. Also, siphonoxanthin, a carotenoid found in siphonous green algae, has been reported to exhibit remarkable bioactivities, related to inducing apoptosis on human leukemia cells and anti-angiogenesis, in our previous studies [6,7,8,9]. Recently, we found that siphonaxanthin possesses anti-obesity effects in KK-Ay mice [10] and exerts anti-inflammatory effects by suppressing antigen-induced degranulation in rat basophilic leukemia cells [11].

**Figure 1:**
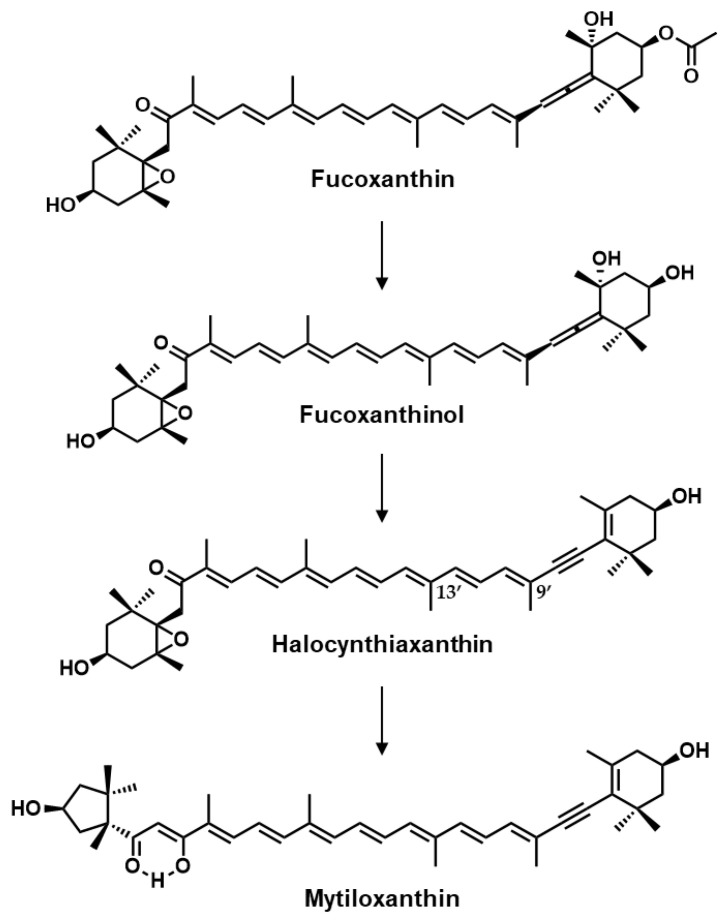
Chemical structures of fucoxanthin metabolites and their metabolic pathway in tunicates.

Halocynthiaxanthin is a carotenoid that can be isolated from sea squirts, mainly *Halocynthia roretzi*, with acetylenic bonds, a keto group, an epoxy group, and a hydroxyl group in its chemical structure (Figure 1) [12,13]. *H. roretzi* is an edible marine organism that is classified into the family Pyuridae in Prochordata and has been consumed in East Asia, especially in South Korea and in the Northeast region of Japan [14]. In previous studies, the pathway by which *H. roretzi* metabolites fucoxanthin, derived from marine phytoplankton, to mytiloxanthin via halocynthiaxanthin and fucoxanthinol has been suggested (Figure 1) [15,16]. The transformation from fucoxanthin to fucoxanthinol also occurs in mice and human small intestinal epithelial cells, although halocynthiaxanthin was not found to be a metabolite [17]. This structurally unique carotenoid exhibits anti-tumor activity [18] and has an induction effect on apoptosis in cancer cells [19]. In addition, the inhibitory effects of halocynthiaxanthin on human herpesvirus type 4 activity and nitric oxide production in mouse macrophage-like cells have been reported [20,21]. We also found that halocynthiaxanthin effectively suppressed ligand-induced immunoreceptor lipid raft translocation, indicating that it could have pleiotropic immunomodulatory effects [22].

In general, dietary carotenoids are ingested and absorbed at several stages. Carotenoids are released from the food matrix and dispersed into emulsions via digestion, and then incorporated into smaller particles, which are called mixed micelles. After they are solubilized in micelles, carotenoids are absorbed from the small intestinal epithelium and transported to the lymph by chylomicrons. Carotenoids are then transferred to the blood and organs, where carotenoids can accumulate and exert functions [23]. Understanding the intestinal absorption and metabolic mechanisms of carotenoids is one of the major goals to examine their bioactivities because the degradation products and metabolites that derive from these processes could also have inherent functions. For instance, the induction effect of acyclo-retinoic acid, an oxidation product of lycopene, on the growth of human prostate cancer cells has been reported [24]. However, the digestion and absorption of halocynthiaxanthin still remain unclear, and its gastrointestinal absorption in mammals has not been studied yet.

In this study, we evaluated the intestinal absorption of halocynthiaxanthin in mice to examine its mechanism of digestion and absorption, aiming to utilize seaweed in the food industry, mainly as a nutraceutical and functional food. This study broadens our understanding of the absorption of dietary carotenoids in the human body.

## 2. Results

**Figure 2:**
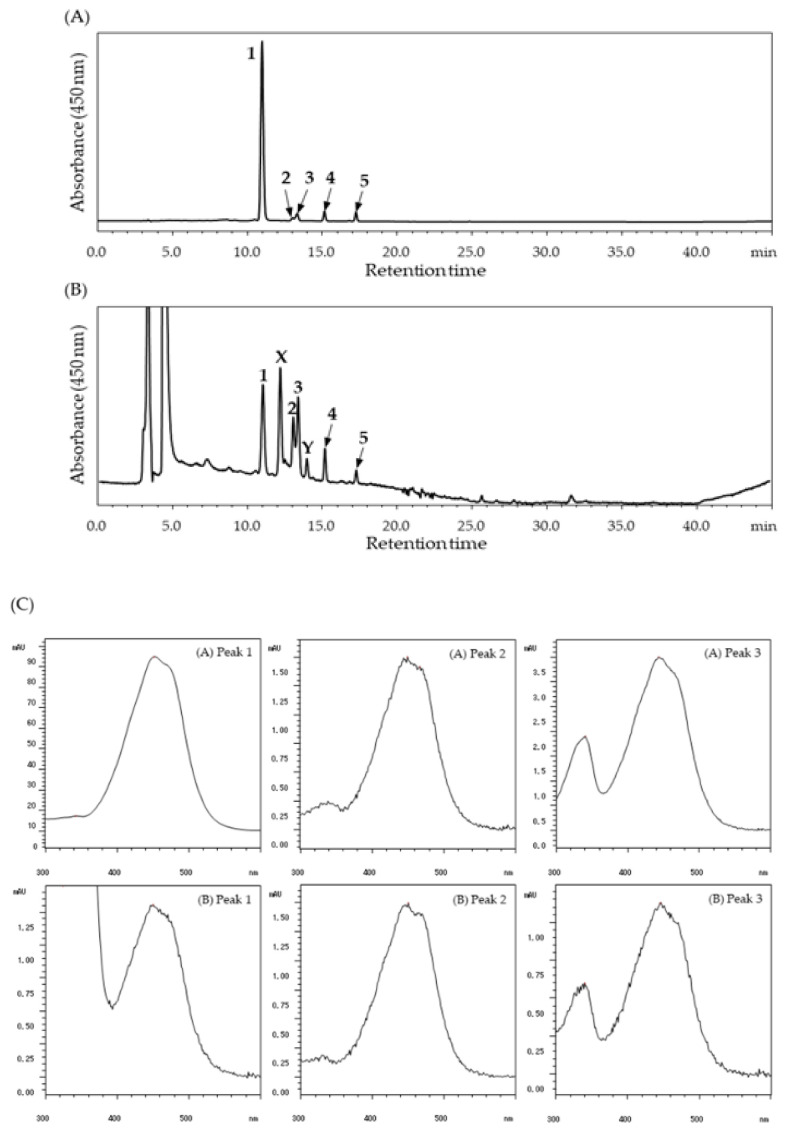
HPLC chromatograms and UV-visible absorption spectra (VIS) for the detection of dietary halocynthiaxanthin. (**A**) Xanthophyll fraction used for oral administration in mice; (**B**) Blood plasma of a mouse 3 h after ingestion of the xanthophyll fraction. The detection wavelength was 450 nm. Peaks: (**1**) all-*trans*-halocynthiaxanthin; (**2**) 9′-*cis*-halocynthiaxanthin; (**3**) 13′-*cis*-halocynthiaxanthin; (**4**) all-*trans*-alloxanthin; (**5**) *cis*-alloxanthin. (**C**) UV-VIS of each halocynthiaxanthin isomer in (A) the xanthophyll fraction and (B) mouse plasma, 3 h after administration.

The halocynthiaxanthin-rich xanthophyll fraction for animal studies consisted mainly of all-*trans*-halocynthiaxanthin (83%) and of a small amount of the 9′-*cis* isomer (2%) and the 13′-*cis* isomer (3%) of halocynthiaxanthin. In addition, all-*trans*-alloxanthin and its *cis*-isomer were detected in the fraction (Figure 2A). All-*trans*-halocynthiaxanthin was detected in the plasma 1 h after administration, while the 9′-*cis* and 13′-*cis* isomers were found at 3 h after ingestion (Figure 2B). These peaks were not found in mouse plasma with no treatment (normal). As shown in Figure 2C, the UV-VIS spectrum of each halocynthiaxanthin isomer (peak 1–3) in mouse plasma after digestion was in agreement with that of the xanthophyll fraction. Interestingly, two peaks (peak *X*, retention time around 12.2 min; peak *Y,* retention time around 14.0 min) were also detected (Figure 2B). Peak *X* and *Y* were not detected in either normal mouse plasma or the halocynthiaxanthin-rich xanthophyll fraction; hence, it is possible that these are metabolites of halocynthiaxanthin.

**Figure 3:**
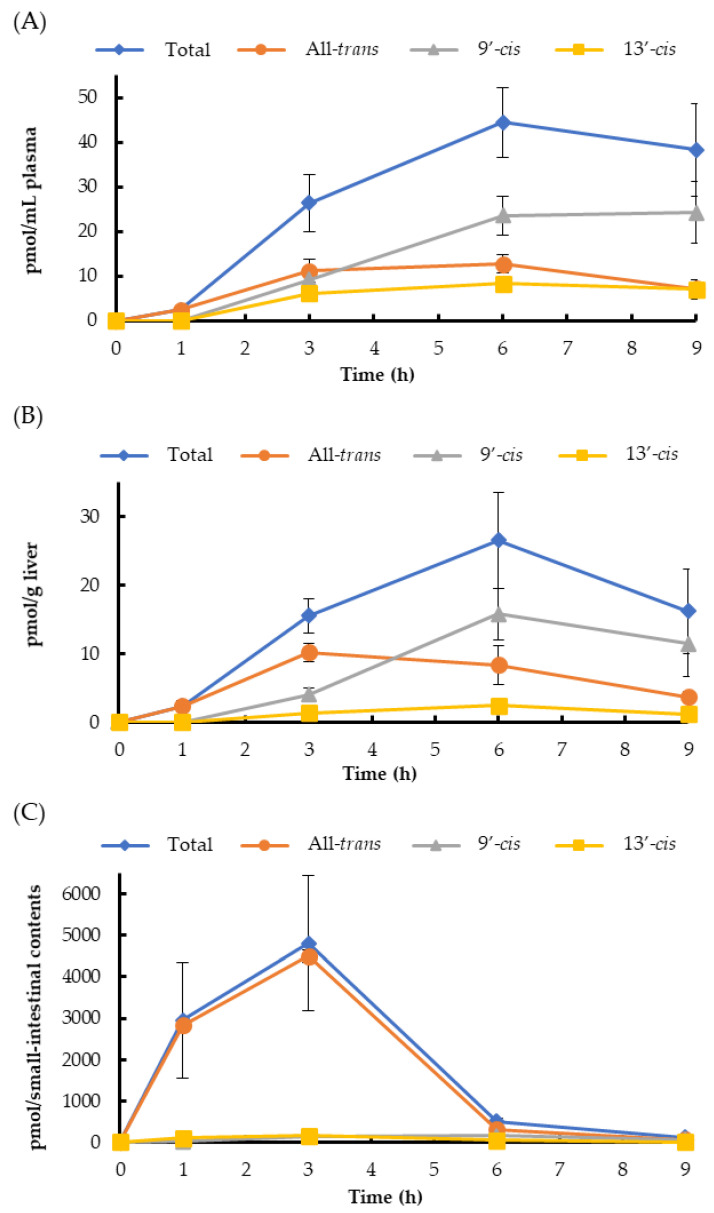
The time course changes in the halocynthiaxanthin isomer levels in mouse blood plasma (**A**), liver (**B**), and small-intestinal contents (**C**). Each value is the mean ± S.E. (*n* = 3 or 4).

The time-course changes of halocynthiaxanthin levels in the plasma and liver showed a similar tendency (Figure 3). The total halocynthiaxanthin increased until 6 h, and all-*trans*-halocynthiaxanthin was the major isomer until 3 h after ingestion. The 13′-*cis* isomer gradually increased until 6 h. The 9′-*cis* isomer clearly increased, especially from 3 h to 6 h after administration (Figure 3A,B). In the small intestine, the total and all-*trans*-halocynthiaxanthin levels increased until 3 h, while those of 9′-*cis* and 13′-*cis* isomers slightly increased until 3 h, unlike the changes observed in the plasma and liver (Figure 3C).

**Figure 4:**
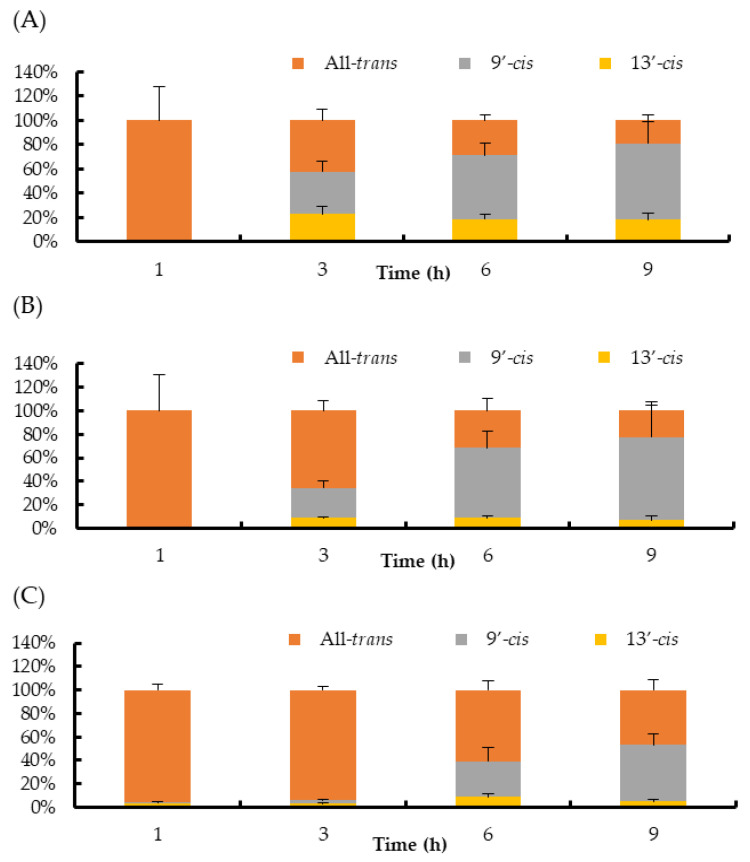
Ratios of halocynthiaxanthin isomers in mouse blood plasma (**A**), liver (**B**), and small intestine (**C**). Each value is the mean ± S.E. (*n* = 3 or 4).

Regarding the isomer composition of halocynthiaxanthin in the plasma and liver, the ratio of the 9′-*cis* isomer increased clearly after 3 h and accounted for more than 50% of the total halocynthiaxanthin after 6 h (Figure 4A,B). In the small intestine, all-*trans*-halocynthiaxanthin remained as the major isomer until 3 h. The ratio of the 9′-*cis* and the 13′-*cis* isomers increased after 6 h. However, the 9′-*cis* isomer did not increase remarkably, unlike it increased in the plasma and liver (Figure 4C).

## 3. Discussion

In this study, we confirmed that dietary halocynthiaxanthin can be absorbed from the intestine and found in the plasma as an all-*trans* and *cis*-isomer. Halocynthiaxanthin is a metabolite of fucoxanthin found mainly in sea squirts; however, mammals cannot convert fucoxanthin to halocynthiaxanthin [17,25,26]. Thus, halocynthiaxanthin needs to be ingested directly by humans in order to take advantage of its biological activities. The bioavailability of dietary halocynthiaxanthin appears to be comparable to that of other carotenoids, depending on the condition of evaluation [17,26,27,28]. 

Interestingly, our results indicated that 9′-*cis*-halocynthiaxanthin was the main isomer present in the plasma and liver after oral ingestion, although the 9′-*cis* isomer is not abundant and exists only in small amounts in the administrated sample. On the other hand, all-*trans*-halocynthiaxanthin was abundant in the small intestine, whereas it was not the case in the liver and plasma, in our experiment. A previous study showed that astaxanthin can be transferred and accumulated in the plasma and organs with selectivity, depending on different isomers. In particular, the 13-*cis* isomer was detected in more remarkable amounts than other isomers in the mouse plasma [28]. This tendency was considerable in the liver, whereas all-*trans* astaxanthin was dominant in the small intestine. Thus, carotenoids are possibly isomerized after absorption in the small intestine, considering the distribution of carotenoid isomers in the plasma, liver, and small intestine. Another possible reason is that the *cis*-isomers may be absorbed easier than the *trans*-isomers. The structure of carotenoids is considered to be a key factor in determining their bioactivity, mechanism of action, and chemical properties [29]. *cis*-lycopene is known to be more soluble in mixed micelles and preferentially incorporated into chylomicrons because it is structurally uneasy to aggregate, compared to the all-*trans* isomer [30]. Therefore, there is a possibility that the *cis*-isomers are absorbed more easily than the all-*trans* isomers due to their chemical structure, especially in the case of symmetrical carotenoids, which are easily aggregated. 

To evaluate the food function of carotenoids, the correlation between isomers and their bioactivities is very important. In fact, the *cis*-isomer of fucoxanthin has shown a stronger inhibition of cancer cell growth than the *trans*-isomer [31]. In addition, *cis*-astaxanthin showed a higher antioxidant activity than the all-*trans* isomer [32]. It has also been reported that *cis*-lycopene isomers exhibit a stronger antioxidant activity than the all-*trans* isomer [30,33]. Based on the result that the 9′-*cis* isomer was major in the mouse plasma and liver, further examination of the difference in bioavailability and bioactivity of each isomer is required. 

Fucoxanthin, a carotenoid found in brown algae, is converted to fucoxanthinol and amarouciaxanthin A, and the metabolism of fucoxanthinol to amarouciaxanthin A progresses mainly in the mouse liver. This transformation has been considered to progress via NAD(P)^+^-dependent dehydrogenase under alkaline conditions (pH 9.5–10.0). The report states that the enzymes that catalyze the conversion would exist in live microsomes [25]. We have also previously reported that three oxidized metabolites were detected, following dietary administration of siphonaxanthin in mice [34]. In the current study, two peaks, *X* and *Y*, which might be metabolites of halocynthixanthin, were detected in the mouse plasma after administration of halocynthiaxanthin (Figure 2). Halocynthiaxanthin has an epoxy group in its molecule and the same structure on the left side, such as fucoxanthin and fucoxanthinol. Hence, the unknown products could be transformed from halocynthiaxanthin via enzymatic reactions. Further studies are needed to identify the unknown products and metabolic pathways of halocynthiaxanthin.

Taken together, our results indicated that dietary halocynthiaxanthin can be absorbed and transferred into the body in mice, and 9′-*cis*-halocynthiaxanthin was the dominant isomer in vivo. In addition, obtaining halocynthiaxanthin directly from food would be important because it has thus far not been reported that mammals can convert it from other carotenoids, such as fucoxanthin. To our knowledge, this is the first report evaluating the intestinal absorption of halocynthiaxanthin in mice.

## 4. Materials and Methods 

### 4.1. Preparation of Halocynthiaxanthin

The halocynthiaxanthin-rich xanthophyll fraction and purified halocynthiaxanthin were prepared following the procedure below, for animal studies and for identification, respectively. The ethanol extract from the dried powder of whole H. roretzi was kindly donated by Nihon Pharmaceutical Co., Ltd. (Tokyo, Japan). The fractions from tunicate sea squirt, *Halocynthia roretzi*, were extracted from the ethanol extract using acetone at room temperature. The acetone extract was partitioned between *n*-hexane-ether (7:3, *v*/*v*) and aqueous NaCl. The organic layer was dried over Na_2_SO_4_ and evaporated. The residue was subjected to silica gel column chromatography. The fraction eluted with acetone from the silica gel column was further separated using preparative HPLC on ODS using CHCl_3_:CH_3_CN (1:9, *v*/*v*) at a flow rate of 2.0 mL/min to yield halocynthiaxanthin. The identification and estimation of the purity of halocynthiaxanthin was carried out using ^1^H-NMR and ESI MS spectral data. We did not observe the production of the 5,8-isomer that could arise due to HCl contained in CHCl_3_.

### 4.2. Animal Studies

All experimental animal protocols were approved by the Animal Experimentation Committee of Kyoto University, Japan, for the care and use of experimental animals (Approval No. 31–37). The halocynthiaxanthin-rich xanthophyll fraction was diluted in olive oil to a final concentration 0.84 µg/µL halocynthiaxanthin. The final concentration was measured after sample preparation and the administration volume was decided based on this concentration and mice body weights. Male ICR mice (6-week-old) were purchased from Japan SLC Inc. (Hamamatsu, Japan). All mice were kept on light-dark cycles (12 h shifts) at a temperature of 24 °C ± 2 °C with free access to AIN-93G diet and water. After an acclimation period of 1 week, mice were randomly divided into a no-treatment control group (*n* = 3) and halocynthiaxanthin groups (1, 3, 6, and 9 h) (*n* = 3 or 4 per group). Only the halocynthixanthin group was orally administered halocynthiaxanthin at a dose of 5 mg/kg body weight. After oral administration (1, 3, 6, and 9 h)**,** mice were anesthetized with isoflurane (Mylan Seiyaku Ltd., Tokyo, Japan). Blood was collected from the inferior vena cava and centrifuged at 2,000 rpm for 15 min at 4 °C to prepare the plasma. Plasma was stored at −80 °C until use. After perfusion with saline through the heart, the liver and small intestine were removed and rinsed with saline. Tissues were immediately weighed, frozen in liquid nitrogen after dissection, and stored at −80 °C. 

### 4.3. Lipid Extraction

For a total lipid extraction from the plasma samples, 0.4 mL of plasma were diluted in 9 volumes of saline and vortexed with 4.4 mL of methanol and 8.8 mL of chloroform for 60 s. After centrifugation (1700× *g*, 15 min, 4 °C), the lower chloroform layer was collected. The same amount of chloroform as the collected chloroform layer was added to the mixture, and this extraction procedure was repeated twice. The combined chloroform layer was dried under a stream of nitrogen and dissolved in a small amount of chloroform/methanol (2:1, *v*/*v*), and finally stored at −80 °C until use. For the lipid extraction from tissues, 200 mg of liver tissue were homogenized with 9 volumes of saline using a Potter-Elvehjem homogenizer. Precisely 1.8 mL of the homogenate were vortexed with 2.0 mL of methanol and 4.0 mL of chloroform for 60 s. The mixture was centrifuged at 1700× *g* for 15 min at 4 °C, and the chloroform layer was collected and stored, as previously described. The small intestinal contents were obtained by washing with approximately 8 mL of ice-cold phosphate-buffered saline. Lipids were extracted from the small intestinal contents after mixing with 8.8 mL of methanol and 17.6 mL of chloroform, based on the same procedure, as previously described.

### 4.4. HPLC Analysis

After evaporation of chloroform/methanol (2:1, *v*/*v*) with nitrogen blowdown, the extracted lipids were suspended in 40 µL of ethyl acetate/methanol (1:1, *v*/*v*), and 25 µL of the suspension was used for HPLC analysis. In the animal studies samples, halocynthixanthin was quantified from the peak area at 450 nm using the calibration curve of purified halocynthixanthin. The HPLC analysis was performed on a TSKgel ODS-80Ts column (4.6 mm × 250 mm, Tosoh, Tokyo, Japan) using a Prominence LC System (Shimadzu, Kyoto, Japan) with a photodiode array detector SPD-M20A (Shimadzu, Kyoto, Japan). For the binary gradient elution, methanol/Milli-Q water (90:10, *v*/*v*) containing 0.1% ammonium acetate was used for mobile phase A, and methanol/ethyl acetate (70:30, *v*/*v*) containing 0.1% ammonium was used for mobile phase B using the following gradient program: 0–5 min, 0% B; 5–20 min, 0–100% B; 20–35 min, 100% B; 35–40 min, 100–0% B; 40–45 min, 100% B, at a flow rate of 1.0 mL/min.
